# Dietary Phenethyl Isothiocyanate Alters Gene Expression in Human Breast Cancer Cells

**DOI:** 10.1155/2011/462525

**Published:** 2010-09-28

**Authors:** Young Jin Moon, Daniel A. Brazeau, Marilyn E. Morris

**Affiliations:** Department of Pharmaceutical Sciences, School of Pharmacy and Pharmaceutical Sciences, University at Buffalo, State University of New York, Amherst, NY 14260, USA

## Abstract

Phenethyl isothiocyanate (PEITC), a component in cruciferous vegetables, can block chemical carcinogenesis in animal models. Our objective was to determine the effect of treatment with PEITC on gene expression changes in MCF-7 human breast cancer cells in order to evaluate potential mechanisms involved in its chemopreventive effects. MCF-7 cells were treated for 48 hours with either PEITC (3 *μ*M) or the vehicle. Total RNA was extracted from cell membrane preparations, and labeled cDNA's representing the mRNA pool were reverse-transcribed directly from total RNA isolated for use in the microarray hybridizations. Two specific human GE Array Kits (Superarray Inc.) that both contain 23 marker genes, related to signal transduction pathways or cancer/tumor suppression, plus 2 housekeeping genes (*β*-actin and GAPDH), were utilized. Arrays from treated and control cells (*n* = 4 per group) were evaluated using a Student's *t*-test. Gene expression was significantly induced for tumor protein p53 (p53), cyclin-dependent kinase inhibitor 1C (p57 Kip2), breast cancer Type 2 early onset (BRCA2), cAMP responsive element binding protein 2 (ATF-2), interleukin 2 (IL-2), heat shock 27 KD protein (hsp27), and CYP19 (aromatase). Induction of p57 Kip2, p53, BRCA2, IL-2, and ATF-2 would be expected to decrease cellular proliferation and increase tumor suppression and/or apoptosis. PEITC treatment produced significant alterations in some genes involved in tumor suppression and cellular proliferation/apoptosis that may be important in explaining the chemopreventive effects of PEITC.

## 1. Introduction

Diet and environmental factors may represent very significant factors in the genesis of breast cancer. Organic isothiocyanates (ITCs, R-N=C=S), also known as mustard oils, are present in the form of glucosinolates in *Brassica* and other vegetables of the family Cruciferae (e.g., cabbage, cauliflower, brussels sprouts, watercress, and broccoli, kale) and the genus Raphanus (radishes and daikons) [1]. When vegetables are ingested, ITCs are liberated through the hydrolysis of glucosinolates either by myrosinase that is released when vegetables are chewed, or by microflora in the intestinal tract [1, 2]. The human intake of glucosinolates, the biological precursors of ITCs, can be as high as 300 mg/day (660 *μ*mol/day) and tens of milligrams of ITCs are released following the consumption of vegetables in the diet [3]. Additionally, dietary supplements containing ITCs or extracts of cruciferous vegetables, such as Cruciferous Plus and Broccoli Sprouts, are marketed for their health promotion properties. 

More than 20 natural and synthetic ITCs have been shown to block chemical carcinogenesis induced by environmental carcinogens including polycyclic aromatic hydrocarbons and nitrosamines in animal models [3–6]. It has been reported that urinary ITC levels are significantly associated with reduced breast cancer risk in pre- and postmenopausal women [7]. One study demonstrated that *Brassica* vegetable consumption increases the ratio of 2-hydroxyestrone (a noncarcinogenic metabolite of estrogen) to 16*α*-hydroxyestrone (a carcinogenic metabolite of estrogen), in healthy postmenopausal women [8]. The mechanisms underlying the chemopreventive effects of ITCs are likely diverse and multi-factorial and remain largely unknown at this time. 

**Figure 1:**
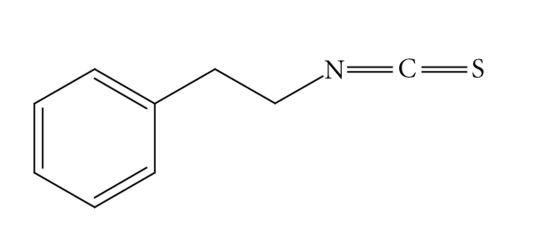
Chemical structure of phenethyl isothiocyanate (PEITC).

Phenethyl isothiocyanate (PEITC, Figure 1) is one of the most extensively studied ITCs; it has been reported to have effective chemopreventive activity for a wide variety of tumors, and no apparent toxicity has been observed in animal models [9]. PEITC can inhibit Phase I enzymes, including the various cytochrome P450 enzymes that are responsible for the conversion of procarcinogens to highly reactive electrophilic carcinogens that can form DNA adducts; PEITC can also induce Phase II enzymes, including glutathione S-transferase and quinone reductase, that inactivate carcinogens and promote their excretion [10, 11]. More recently, it has been found that PEITC could induce cell cycle arrest and apoptotic cell death [12–17]. The IC_50_ value of PEITC for inhibition of cell growth of human breast cancer MCF-7 cells (evaluated over a 48-h period) is 6.51 ± 0.86 *μ*M [16]. Interestingly, PEITC acts more potently than the pure antiestrogen ICI 182,780 (Fulvestrant; Faslodex) to inhibit the growth of estrogen receptor positive breast cancer MCF-7 and H3396 cells and ER-negative MDA-MB-231 and SK-BR-3 cells [17]. In addition, PEITC, but not ICI 182,780, can downregulate the steady state levels of ER-*α*36 protein (36 kDa variant of the ER-*α*, which mediates membrane-initiated estrogen and antiestrogen signaling) in breast cancer cells [17]. The exact mechanism underlying the effect of PEITC on the ER-*α*36 protein is unknown [17]. PEITC is known to selectively kill cancer cells, but not normal cells, by generating reactive oxygen species (ROS) to trigger signal transduction, resulting in cell cycle arrest and/or apoptosis [18]. PEITC is also an effective inhibitor of hypoxia inducible factor (HIF), a transcription factor that plays an important role in the expression of proangiogenic factors [19].

To better understand the precise molecular mechanism(s) by which PEITC exerts its effects on MCF-7 human breast cancer cells, we utilized cDNA gene arrays to assess the gene expression profiles of breast cancer cells treated with physiologically relevant levels (3 *μ*M) of PEITC. Studies have examined the mechanisms underlying the cell growth inhibitory effects of PEITC in human leukemia [20], lung cancer [21], HeLa cervical cancer [22], HT-29 colon adenocarcinoma [15], pancreatic cancer [23], and human prostate cancer PC-3 [24] cells. However, few studies have used breast cancer cells for investigating the effect of PEITC on gene expression. In the present study, genes involved in cell cycle pathways, apoptosis, and metastasis, important for chemoprevention, were evaluated by gene-array expression technology. 

## 2. Methods

### 2.1. Materials

PEITC and dimethyl sulfoxide (DMSO) were purchased from Sigma-Aldrich (St. Louis, MO). RPMI 1640 medium, fetal bovine serum, and PBS were from Invitrogen (Carlsbad, CA). MCF-7 cells were obtained from the National Cancer Institute. 

### 2.2. Cell Culture

MCF-7 cells were grown in 75 cm^2^ cell culture flasks in RPMI 1640 culture media supplemented with 10% FBS, 100 units/ml penicillin and 100 *μ*g/ml of streptomycin, in a 37°C incubator in a humidified atmosphere of 5% CO_2_/95% air. MCF-7 cells, with passage numbers of 16 to 24, were used in the experiments. Cells were treated with either 3 *μ*M PEITC or 0.015% DMSO (vehicle control) for 48 h. The rationale for choosing this time point was to capture gene expression profiles of genes involved during the onset of growth inhibition and apoptotic processes. The concentration of PEITC used is one that is achievable in plasma after the consumption of food or dietary supplements. Plasma concentration of up to 1 *μ*M has been reported after the ingestion of watercress 100 g [25].

### 2.3. Total RNA Isolation

Total RNA from each sample was isolated using a SV Total RNA Isolation System (Promega, Cat.#Z3100), following the manufacturer's instructions. Total RNA was quantitated spectrophotometrically at 260 nm. 

### 2.4. Gene Array

Two specific human GEArray Kits (SuperArray Inc., Frederick, MD, signal transduction pathway array and cancer/tumor suppressor array), were utilized. Each array consists of 23 genes in duplicate, as well as control spots (PUC18 as negative control; *β* actin and glyceraldehyde-3-phosphate dehydrogenase (G3PDH)) (Table 1). The gene arrays were used according to the manufacturer's instructions. In brief, using the reagents provided (i.e., 5X GEAlabeling Buffer, RNase-free water, RT Primer, RNase Inhibitor, and the array-specific reagent), gene specific cDNAs were prepared and labeled from total RNA by reverse transcription with MMLV reverse transcriptase (Invitrogen, Carlsbad, CA) and chemiluminescence-labeled biotin dUTP (Invitrogen, Carlsbad, CA). Relative expression levels of each gene were analyzed using a Kodak Image Station 440CF. *β* actin was used for normalization. Each experiment was repeated four times. 

### 2.5. Real-Time Quantitative Reverse Transcriptase-Polymerase Chain Reaction (RTQ RT-PCR)

Real-time quantitative reverse transcriptase polymerase chain reaction was performed on *β* actin (for normalization) and CYP19 using Stratagene's Mx4000 Multiplex Quantitative PCR System (Stratagene, La Jolla, CA). The same total RNA prepared for the gene arrays was also used for RTQ RT-PCR. Total RNA (560 ng) from each sample was reverse transcribed into cDNA using a Superscript first strand cDNA synthesis kit (Invitrogen, Carlsbad, CA) according to the manufacturer's protocol. PCR reactions for CYP19 and *β* actin were carried out by mixing 5 *μ*L of cDNA, 5 *μ*L of 10  × PCR buffer, 2 *μ*L of deoxynucleoside triphosphate mix (5 mM each dATP, dCTP, dGTP, and dTTP), 1 *μ*L each of 10 *μ*M primer, 0.5 *μ*L reference dye rhodamine-X (1/500 dilution, Molecular Probes, Eugene, OR), 0.5 *μ*L SYBR green I (1/750 dilution, Molecular Probes, Eugene, Or), 2 U Taq polymerase (Eppendorf, Westbury, NY) and 34.75 *μ*L H_2_O, and amplified for 40 cycles. Primers for *β* actin (forward: 5′-CTGGCCGGGACCTGACT-3′, Reverse: 5′- TCCTTAATGTCACGCACGATTT-3′, annealing temperature: 57°C) were designed using the computer program Primer Express (Perkin-Elmer Applied Biosystems, Foster City, CA). Primers for CYP19 (Forward: 5′-TGGAAAACAACTCGACCCTTCT-3′, Reverse: 5′-CACAGACTGTGACCATACGAACAA-3′) have been described [26]. The PCR products were resolved by electrophoresis through a 2% agarose gel to confirm target size and the presence of single PCR product. 

The PCR product of each gene was cloned into a pCR 2.1 TOPO vector (Invitrogen, Carlsbad, CA) and transformed into One Shot chemically competent *Escherichia coli* cells (Invitrogen, Carlsbad, CA). Cloned PCR products were confirmed by sequencing and used to construct standard curves for absolute quantification of copy number. The standard curves were run in triplicate concurrently on the same plate with samples, which were also run in triplicate. The reported copy number was estimated from the linear regression of the standard curve on the same plate. 

### 2.6. Statistical Analysis

Student's *t*-tests with *P* < .05 was used for statistical analysis for both arrays and RTQ RT-PCR.

## 3. Results

**Figure 2:**
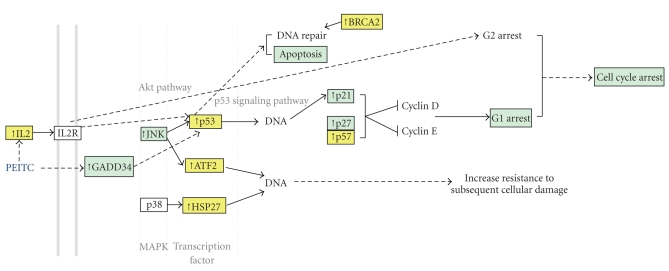
Significantly altered genes by PEITC from the current study are presented in yellow boxes, and the effects of PEITC that have already been reported in previous studies are presented in blue boxes. All genes (ATF-2, p53, Hsp27, p57, BRCA2, IL-2) were upregulated. ATF-2, p53, Hsp27 are transcription factors which are all located downstream of MAPK signaling pathway (http://david.abcc.ncifcrf.gov/). Induction of ATF-2, p53 and Hsp27 in response to various stresses correlates with increased resistance to subsequent cellular damage [27]. Transcriptional activation of the p53 target genes plays a critical role in the cellular response to DNA damage, cellular stress and other signals regulating the cell cycle and apoptosis [28, 29]. IL-2 induces G2 cell cycle arrest via Akt pathway [30]. BRCA2 is essential for the maintenance of genetic stability through a function in DNA repair [31].

Treatment of MCF-7 cells for 48 hours with PEITC (3 *μ*M) significantly altered the expression of seven of the 46 genes present on the two arrays: p53, ATF-2, hsp27, BRCA2, IL-2, p57, and CYP19 (Table 2 and Figure 2). All 7 genes were upregulated by ~2- to 5-fold. Alterations in 5 of these genes (p53, ATF2, BRCA2, IL-2, and p57) could be considered to have beneficial effects for cancer prevention. A listing of genes tested using Superarrays (Cancer/Tumor suppressor array and Signal transduction pathway array) can be found in Table 1.

## 4. Discussion

PEITC increased the expression of p53, a tumor suppressor gene, by 2.12 fold [32]. Tumor suppressor genes encode for proteins whose normal function is to inhibit cell transformation and whose inactivation results in tumor cell growth and survival [32]. Located on chromosome band 17p13, p53 encodes a 53-kd multifunctional transcription factor that regulates the expression of genes involved in cell cycle control, apoptosis, DNA repair, and angiogenesis [32]. Induction of p53 by PEITC at a low concentrations (≤10 *μ*M) is consistent with the findings of a publication that reported the effects of PEITC in human nonsmall cell lung carcinoma A549 cells [33]. In these cells, induction of apoptosis occurred at low concentrations of PEITC (≤10 *μ*M), and Western blot analyses demonstrated that increased expression of p53 protein was associated with PEITC-induced apoptosis [33]. c-Jun N-terminal kinase (JNK) is involved in PEITC-induced apoptosis, and p53 is known as a substrate of JNK [34]. In contrast, recent immunoblot analyses showed that PEITC 3 *μ*M did not increase the protein expression of p53 in MCF-7 cells [35]. However, the shorter incubation times (12, 24, and 36 hours) used in this study may not have been long enough to produce changes in protein expression. The treatment of MCF-7 cells with PEITC (10 or 30 *μ*M for 24 or 36 h) suppressed the expression of p53 [35], suggesting that the effect of PEITC on p53 expression may be dose- and time-dependent.

Although we did not investigate the effect of PEITC on p53 activity, it is possible that PEITC also induces p53 activity through GADD34 (Growth Arrest and DNA Damage-Inducible Protein), a proapoptotic gene. In a previous study, 25 *μ*M PEITC upregulated expression of GADD34 mRNA [36], and GADD34 is known to induce p53 phosphorylation [37]. In fact, it has recently been reported that PEITC can selectively deplete mutant p53 and restore wild type function to p53 in a variety of tumor cells [38]. In this study, tumor cell lines with mutant p53 became more sensitive to PEITC-induced cytotoxicity than tumor cells with wild type p53, suggesting that the normal p53 checkpoint control pathways have been restored in the mutant p53-expressing tumor cells.

Expression of ATF-2 was increased by PEITC (2.60-fold). ATF-2 is a member of the ATF/CREB family of proteins. It is also known as a cAMP response element-binding protein (CREBP-1). It acts as a transcription factor which regulates transcription in response to the extracellular signals and has a decisive role in cell proliferation, tumorigenesis and apoptosis. Breast cancer frequently develops in mutant mice heterozygous for the ATF-2 gene [39]. Therefore, the ATF-2 gene is considered as a candidate tumor suppressor gene [39]. Changes in gene expression may be mediated through the mitogen-activated protein kinase (MAPK) pathway. PEITC can regulate MAPK—mediated luciferase reporter gene activities [40]. MAPKs can phosphorylate many transcription factors, including c-Jun and ATF-2, and ultimately lead to changes in gene expression [40].

PEITC exposure resulted in the upregulation of Hsp27 by 1.84-fold in MCF-7 cells in this study. The induction of Hsp27 in response to various types of stress correlates with increased resistance to subsequent cellular damage [27]. Hsp27 has also been reported to inhibit apoptosis in NF*κ*B and p53 signaling pathways [41]. However, activation of Hsp27 by PEITC may not result in inhibition of apoptosis, as reported for PEITC-treated human hepatoma HepG2 cells [41].

p57 is a tight-binding inhibitor of several G1 cyclin/Cdk complexes and a negative regulator of cell proliferation [42]. Even though information is lacking regarding the effect of PEITC on p57 gene expression, there are several studies which have shown that PEITC can induce other regulators of cell cycle progression at G1, such as p21WAF-1/Cip-1 and p27Kip1, resulting in a cell-cycle arrest in the G1-phase in vascular smooth muscle cells in vitro [43], and prostate cancer cells in xenograft mice [44]. PEITC can also induce G1 cell cycle arrest on HT-29 cells [45]. Mutations of this gene are implicated in sporadic cancers and Beckwith-Wiedemann syndrome, suggesting that this gene is a tumor suppressor candidate [46].

PEITC upregulated BRCA2 by 4.22-fold in the current study. BRCA2 functions as a tumor suppressor gene which stabilizes DNA structures at stalled replication forks [47]. Mutations in BRCA2 have been linked to an elevated risk of breast cancer in young women, which has been demonstrated to be due to the inheritance of dominant susceptibility genes conferring a high risk of breast cancer. An impaired cellular response to DNA damage appears to be a plausible mechanism by which BRCA carriers are at an increased risk of breast cancer [47].

Interleukin-2 (IL-2) was upregulated by PEITC in this investigation. IL-2 is a cytokine produced by T cells whose main function is to stimulate the growth and cytotoxic response of activated T lymphocytes [48]. IL-2 has been used to stimulate the immune system for the treatment of a number of different tumors, including breast cancer [48]. Foa et al. [49] reported that the constitutive secretion of IL-2 by tumor cells led to a reduced or abrogated tumorigenicity in several different tumor models [49]. IL-2 also induces G2 cell cycle arrest via the Akt pathway [30]. However, other studies have suggested that IL-2 therapy may stimulate tumor cell growth. For example, a short 2-day treatment of low-dose IL-2 resulted in a decrease in tumor load and an increase in survival, whereas the longer administration of IL-2 promoted CD8 T cell growth [50]. Also, the addition of IL-2 to cyclophosphamide therapy reversed the growth inhibitory effects of cyclophosphamide on B16 melanoma cells and decreased survival time, compared with treatment with cyclophosphamide alone [51]. Therefore, IL-2 may have concentration/time-dependent effects on tumor growth and cytotoxicity.

The gene CYP19 expression was significantly increased by PEITC exposure. We confirmed the upregulation of CYP19 observed in the gene array studies, using RTQ RT-PCR. After normalization to *β* actin, the increase of CYP19 was 1.8-fold compared to controls (*P* = .060) by RTQ RT-PCR, compared to 5.22-fold from the gene array data. The AP-1 motif, which regulates the CYP19 promoter [52], may be involved in the CYP19 induction, because 5–10 *μ*M PEITC has been reported to activate AP-1 activity in both prostate and bladder cancer cell lines [53, 54].

The induction of CYP19 (aromatase) which is a key enzyme involved in the conversion of androgens to estrogens, would be considered a negative effect with regards to cancer prevention [55]. Since estrogen causes cellular proliferation and some estrogen metabolites are considered carcinogens, local expression of aromatase has been correlated with tumor initiation and progression [55]. However, the significance of these results is unknown at this time since it is not known if increased transcription of the aromatase gene results in increased activity. Further studies concerning the effect of PEITC on CYP19 enzyme activity are needed.

## 5. Conclusions

In conclusion, PEITC treatment (3 *μ*M) can produce significant alterations in genes involved in tumor suppression and cellular proliferation/apoptosis. We report for the first time transcriptional induction of p53, ATF-2, hsp27, BRCA2, IL-2, p57, and CYP19 by PEITC in the human breast cancer cells, and our results are supported by other studies which have reported increased expression [33] and increased activity [38] of p53 protein and activation of Hsp27 [41]. These alterations in gene expression may be important in the mechanism of action of PEITC, especially for its chemopreventive effects. Further study is needed to capture some of the early response genes. In addition, the overall effect elicited by beneficial and adverse transcriptional changes needs to be considered. The present study may be useful as a first step in understanding the mechanisms underlying the breast cancer preventive activities of PEITC.

## Figures and Tables

**Table:** (a) Cancer/tumor suppressor array

APC	Adenomatosis polyposis coli
BRCA1	Breast cancer 1, early onset
BRCA2	Breast cancer 2, early onset
CBP	Human CREB-binding protein
DPC4	Human homozygous deletion target in pancreatic carcinoma (DPC4)
IRF-1	Interferon regulatory factor 1
MSH2	mutS (E. coli) homolog 2 (colon cancer, nonpolyposis type 1)
NF2	Neurofibromin 2 (bilateral acoustic neuroma)
p18 (cdk4 inhibitor)	Cyclin-dependent kinase inhibitor 2C (p18, inhibits CDK4)
p19Ink4d	Cyclin-dependent kinase inhibitor 2D (p19, inhibits CDK4)
p21Waf1 (p21Cip1)	Cyclin-dependent kinase inhibitor 1A (p21, Cip1)
p27Kip1	Cyclin-dependent kinase inhibitor 1B (p27, Kip1)
p300	CREB-binding protein
p53	Tumor protein p53 (Li-Fraumeni syndrome)
p57Kip2	Cyclin-dependent kinase inhibitor 1C (p57, Kip2)
PTEN	Phosphatase and tensin homolog (mutated in multiple advanced cancers 1
Rb	Retinoblastoma 1 (including osteosarcoma)
TGFbR1 (ALK-5)	Transforming growth factor, beta receptor I (activin A receptor type II-like kinase, 53 kD)
TGFbR2	Transforming growth factor, beta receptor II (70–80 kD)
TSC-1	Tuberous sclerosis 1
TSC-2	Tuberous sclerosis 2
VHL	Von Hippel-Lindau syndrome
WT1	Wilms tumor 1

**Table:** (b) Signal transduction pathway array

ATF-2 (creb-2)	cAMP responsive element binding protein 2
bax	BCL2-associated X protein
CD5	T-cell surface glycoprotein CD5
c-fos	Human cellular oncogene c-fos
c-myc	v-myc avian myelocytomatosis viral oncogene homolog
CYP19 (aromatase p450) (p450XIX)	cytochrome P450, subfamily XIX (aromatization of androgens)
egr-1	Early growth response 1
Fas (Apo-1) (CD95)	Tumor necrosis factor receptor superfamily, member 6
gadd45	DNA-damage-inducible transcript 1
hsf1 (tcf5)	HSF1 (TCF5, Heat shock factor protein)
hsp27 (hsp b1)	Heat shock 27 KD protein
hsp90 (CDw52)	Hsp90 (Human mRNA for 90-kDa heat-shock protein)
I*κ*B*α* (mad3)	Nuclear factor of kappa light polypeptide gene enhancer in B-cells inhibitor, alpha
IL-2	Interleukin 2
iNOS	Inducible nitric oxide synthase (NOS)
mdm2	Mouse double minute 2, human homolog of; p53-binding protein
NF*κ*B	Nuclear factor of kappa light polypeptide gene enhancer in B-cells 1 (p105)
p19^INK4d^	Cyclin-dependent kinase inhibitor 2D (p19, inhibits CDK4)
p21^Waf1^ (p21^Cip1^)	Cyclin-dependent kinase inhibitor 1A (p21, Cip1)
p53	Tumor protein p53 (Li-Fraumeni syndrome)
p57^Kip2^	Cyclin-dependent kinase inhibitor 1C (p57, Kip2)
pig7	LPS-induced TNF-alpha factor
pig8	etoposide-induced mRNA

**Table 2:** Significantly changed genes following treatment with 3 *μ*M of PEITC (*n* = 4, *P < .05, ***P < .001). Results are expressed as fold change as compared to vehicle controls and represent the average of five independent microarray experiments.

Gene name	Description	Fold ± S.D.
p57	cyclin-dependent kinase inhibitor 1C	5.23*± 2.74
CYP19	cytochrome P450, subfamily XIX	5.22***± 0.988
BRCA2	Breast Cancer 2, early-onset	4.22*± 2.30
IL-2	Interleukin-2, T-cell growth factor	3.16*± 1.22
ATF2	Activating Transcription Factor 2	2.60*± 0.900
p53	Tumor protein p53	2.12*± 1.46
Hsp27	Heat shock 27 KD protein	1.84*± 0.409

## Abbreviations

PEITC: Phenethyl Isothiocyanate
DMSO: Dimethyl sulfoxide
MMLV: Moloney murine leukemia virus
CYP: Cytochrome P450
MAPK: Mitogen-activated protein kinase
IL-2: Interleukin-2.
